# Differing trends in the association between obesity and self-reported health in Portugal and Switzerland. Data from national health surveys 1992–2007

**DOI:** 10.1186/1471-2458-12-588

**Published:** 2012-08-01

**Authors:** Pedro Marques-Vidal, Paula Ravasco, Fred Paccaud

**Affiliations:** 1 Institute of Social and Preventive Medicine (IUMSP), CHUV and Faculty of biology and medicine, Lausanne, Switzerland; 2 Unidade de Nutrição e Metabolismo, Instituto de Medicina Molecular, Faculdade de Medicina da Universidade de Lisboa, Lisbon, Portugal

**Keywords:** Obesity, Self-rated health, Population survey, Trends, Portugal, Switzerland

## Abstract

**Background:**

The escalating prevalence of obesity might prompt obese subjects to consider themselves as normal, as this condition is gradually becoming as frequent as normal weight. In this study, we aimed to assess the trends in the associations between obesity and self-rated health in two countries.

**Methods:**

Data from the Portuguese (years 1995–6, 1998–6 and 2005–6) and Swiss (1992–3, 1997, 2002 and 2007) National Health Surveys were used, corresponding to more than 130,000 adults (64,793 for Portugal and 65,829 for Switzerland). Body mass index and self-rated health were derived from self-reported data.

**Results:**

Obesity levels were higher in Portugal (17.5% in 2005–6 *vs.* 8.9% in 2007 in Switzerland, p < 0.001) and increased in both countries. The prevalence of participants rating their health as “bad” or “very bad” was higher in Portugal than in Switzerland (21.8% in 2005–6 *vs* 3.9% in 2007, p < 0.001). In both countries, obese participants rated more frequently their health as “bad” or “very bad” than participants with regular weight. In Switzerland, the prevalence of “bad” or “very bad” rates among obese participants, increased from 6.5% in 1992–3 to 9.8% in 2007, while in Portugal it decreased from 41.3% to 32.3%. After multivariate adjustment, the odds ratio (OR) of stating one self’s health as “bad” or “very bad” among obese relative to normal weight participants, almost doubled in Switzerland: from 1.38 (95% confidence interval, CI: 1.01–1.87) in 1992–3 to 2.64 (95% CI: 2.14–3.26) in 2007, and similar findings were obtained after sample weighting. Conversely, no such trend was found in Portugal: 1.35 (95% CI: 1.23–1.48) in 1995–6 and 1.52 (95% CI: 1.37–1.70) in 2005–6.

**Conclusion:**

Obesity is increasing in Switzerland and Portugal. Obesity is increasingly associated with poorer self-health ratings in Switzerland but not in Portugal.

## Background

Obesity has reached pandemic proportions [1,2], carries a significant health and economic burden [3,4] and is associated with an increased mortality [5]. In order to be effective, public health campaigns against excess weight have to raise the awareness of the target population that obesity is an “unhealthy” condition, thus prompting adequate management procedures. Still, the association between self-rated health and obesity status is not straightforward. Several studies showed that obesity is associated with a lower self-rated health [6,7], while other studies failed to find such a relationship [8,9].

A recent study [10] showed no particular trends in the association between obesity and self-reported overall health in the USA. One explanation provided by the authors was that obesity was becoming increasingly “normal” (*i.e.* frequent) in the USA, a concept also suggested elsewhere [11]. This would make obese subjects feel they have a “normal”, healthy status when comparing themselves against an increasingly obese population. Still, whether this “normalization” by high obesity prevalence is true remains to be assessed.

Hence, in this study, we used data from National Health surveys to assess the association between obesity and self-reported health according to gender and age group in Portugal and Switzerland, two small European countries with a differing prevalence of obesity [1,12,13]. We also assessed the trends in the association between obesity and self-reported health for the period 1995–2005 (for Portugal) and 1992–2007 (for Switzerland).

## Methods

### Portuguese health survey

Portuguese data were provided upon request by the National Health Observatory ( http://www.onsa.pt) (1995–6 and 1998–9) and the National Institute of Statistics ( http://www.ine.pt) (2005–6). The methodology of the Portuguese national interview surveys has been described previously [12,14]. Briefly, the National Health Surveys were conducted between May 1995 and April 1996 (1995–6), October 1998 and September 1999 (1998–9) and February 2005 and January 2006 (2005–6). The sampling frame was built on census data and included all subjects living in individual housing during that period (collective housing such as hospitals, prisons, military barracks, or retirement houses was excluded). The sample was considered representative of the main regions of continental Portugal (North, Center, Lisbon region, Alentejo, and Algarve). In the 2005–6 survey the autonomous regions of the Azores and Madeira were also included, but not considered in the present analysis. The primary sampling unit (PSU) was the house, and data were derived from the population and housing census. Within each main region, two strata were defined: the freguesias (corresponding to counties) and, within the freguesias, geographically defined units of ±300 lodgings (240 in 2005–6). The PSUs were then randomly selected within each geographically defined unit. All subjects living in the sampling unit (house) were surveyed. All surveys were carried out in compliance with the Helsinki Declaration. Data were collected using face-to-face interviews by trained staff according to a standardized protocol [14], and quality control was maintained by reapplying (by a different interviewer) the same questionnaire to 10% of the initial sample. Participation rates (defined as the percentage of households who responded) as reported by the National Institute of Statistics were 88% in 1995–6, 82% in 1998–9 and 76% in 2005–6.

### Swiss health survey

Data from the four Swiss Health Surveys (SHS) were obtained from the Swiss federal bureau of statistics ( http://www.bfs.admin.ch). The SHS is a cross–sectional, nationwide, population–based telephone survey conducted every 5 years since 1992 by the Federal Statistical Office of Switzerland under a mandate from the federal government [15]. To date, the survey has been carried out four times, in 1992-3, 1997, 2002 and 2007 and can be considered as representative of the Swiss population. The study population was chosen by stratified random sampling of a database of all private Swiss households with fixed-line telephones (as opposed to mobile phones). The first sampling stratum consisted of the seven main regions: West “Leman”, West–Central “Mittelland”, Northwest, Zurich, North–Eastern, Central and South. The second stratum consisted of the cantons, and the number of households drawn was proportional to the population of the canton. In some cantons, oversampling of households was performed to obtain accurate cantonal estimates. The third stratum consisted of the household. One member of the household was randomly selected in advance within all members aged 15 years and over. A letter inviting this selected household member to participate in the survey was sent to each sampled subject, who was contacted thereafter by phone and interviewed using computer–assisted telephone interview (CATI) software to manage dialling and data collection. Face–to–face interviews were organised for subjects older than 75 years. In the case of long–term absence of a sampled subject, a proxy interviewee was requested to provide answers on behalf of the pre-defined sampled person (approximately 3% of households). The interviews were carried out in German, French or Italian, as appropriate. People who did not speak any of these three languages were excluded from the survey. Other criteria for exclusion were asylum seeker status, households without a fixed-line telephone, very poor health status and living in a nursing home [16]. The participation rate was 71% in 1992-3, 85% in 1997, 64% in 2002, and 66% in 2007. It is estimated that <2% of households were excluded owing to these exclusion criteria. Details are available at http://www.bfs.admin.ch/bfs/portal/fr/index/infothek/erhebungen__quellen/blank/blank/ess/01.html.

### Data collected

In both countries, height and weight were self-reported. Body mass index (BMI) was calculated and three categories were defined: normal (BMI < 25 kg/m^2^), overweight (25 ≤ BMI < 30 kg/m^2^) and obese (BMI ≥ 30 kg/m^2^). Self-reported health was coded as “very good”, “good”, “fair”, bad” and “very bad”. Due to the small number of “very bad” answers, we grouped the answers “bad” and “very bad” and performed the multivariate analysis on this pooled group.

### Statistical analysis

Statistical analysis was conducted using SAS v.9.2 (SAS Inc., Cary, NC, USA). Quantitative variables were expressed as mean ± standard deviation and qualitative variables as number of participants and (percentage). Bivariate comparisons were performed using Student *t*-test or a chi-square test for quantitative and qualitative variables, respectively. Multivariate analysis was performed for each country separately using logistic regression adjusting for gender, age, education, smoking status and geographic region. As several studies have reported differing associations between obesity status and self-rated health according to gender and age [17,18], a similar analysis was conducted for each country stratifying on gender and age groups and adjusting for the remaining variables. Results were expressed as odds ratio (OR) and 95% confidence interval (CI). For Switzerland, a further adjustment on nationality was performed as it has been shown that prevalence of obesity differs according to country of origin [19]. Conversely, it was not possible to restrict the analysis to migrants as performed by others [8] due to small sample sizes. Finally, contrary to another study [10], no adjustment for race was performed as data collection regarding race or ethnicity is forbidden by law in both countries. Statistical significance was considered for p < 0.05.

## Results

### Characteristics of the samples

Overall, data from over 120,000 adult participants was analyzed; 64,793 for Portugal and 65,829 for Switzerland. The main clinical characteristics of the samples according to country and study year are summarized in Table 1. In both countries, mean BMI and the prevalence of obesity increased with study year; an improvement in educational level was also noted. The prevalence of current smokers remained stable in Portugal and decreased in Switzerland. Self-rated health improved in Portugal, while in Switzerland the prevalence of participants rating their health as “very good” decreased, although the prevalence of participants rating their health as “good” or “very good” remained relatively stable (Table 1). Of interest, the prevalence of participants rating their health as “bad” or “very bad” was much higher in Portugal (21.8% in 2005–6 *vs.* 3.9% in 2007 for Switzerland).

**Table 1:** Clinical characteristics of the participants, by country and survey year

**Country**	**Survey 1**	**Survey 2**	**Survey 3**	**Survey 4**	**Test**
**Portugal (survey year)**	**1995-6**	**1998-9**	**2005-6**		
Sample size	22,162	22,695	16,899		
Women (%)	13,522 (61.0)	14,284 (62.9)	9923 (58.7)		72.65 ***
BMI (kg/m^2^)	25.6 ± 4.1	25.9 ± 4.2	26.2 ± 4.4		82.07 ***
BMI categories (%)					
Normal	10,582 (47.8)	10,477 (46.2)	7293 (43.2)		
Overweight	8512 (38.4)	8776 (38.7)	6655 (39.4)		129.5 ***
Obese	3068 (13.8)	3442 (15.2)	2951 (17.5)		
Educational level (%)					
Basic	17,611 (79.4)	16,895 (74.4)	11,216 (66.4)		
Secondary	3249 (14.7)	4136 (18.2)	3965 (23.4)		863.4 ***
University	1302 (5.9)	1664 (7.4)	1718 (10.2)		
Smokers (%)	3189 (14.4)	3379 (14.9)	2525 (14.9)		3.10 NS
Self-rated health (%)					
Very good	531 (2.4)	544 (2.4)	963 (5.7)		
Good	5567 (25.1)	6159 (27.1)	5291 (31.3)		821.0 ***
Fair	9385 (42.4)	9920 (43.7)	6965 (41.2)		
Bad	5232 (23.6)	4810 (21.2)	2793 (16.5)		
Very bad	1447 (6.5)	1262 (5.6)	887 (5.3)		
**Switzerland (survey year)**	**1992-3**	**1997**	**2002**	**2007**	
Sample size	14,521	12,470	18,904	17,869	
Women (%)	7946 (54.7)	6934 (55.6)	10,343 (54.7)	9857 (55.2)	3.12 NS
BMI (kg/m^2^)	23.6 ± 3.8	24.0 ± 4.0	24.4 ± 4.1	24.5 ± 4.1	142.9 ***
BMI categories (%)					
Normal	10,091 (69.5)	8148 (65.3)	11,567 (61.2)	10,822 (60.6)	
Overweight	3627 (25.0)	3448 (27.7)	5747 (30.4)	5459 (30.6)	386.0 ***
Obese	803 (5.5)	874 (7.0)	1590 (8.4)	1588 (8.9)	
Educational level (%)					
Basic	3081 (21.2)	2745 (22.0)	3632 (19.2)	2469 (13.8)	
Secondary	8300 (57.2)	7576 (60.8)	12,081 (63.9)	10,518 (58.9)	1004.7 ***
University	3140 (21.6)	2149 (17.2)	3191 (16.9)	4882 (27.3)	
Smokers (%)	4648 (32.0)	4209 (33.8)	5770 (30.5)	4910 (27.5)	153.9 ***
Self-rated health (%)					
Very good	4040 (27.8)	3270 (26.2)	4262 (22.6)	3517 (19.7)	
Good	8127 (56.0)	7076 (56.7)	11729 (62.1)	11,648 (65.2)	
Fair	1782 (12.3)	1612 (12.9)	2224 (11.8)	2005 (11.2)	449.1 ***
Bad	488 (3.4)	419 (3.4)	586 (3.1)	578 (3.2)	
Very bad	84 (0.6)	93 (0.8)	103 (0.5)	121 (0.7)	

Results are expressed as mean ± standard deviation or as number of subjects and (percentage). Statistical analysis by analysis of variance or chi-square: NS, not significant; *, p < 0.05; **, p < 0.01; ***, p < 0.001.

### Association between obesity and self-rated health

On bivariate analyses and after stratifying for study, obese subjects rated their health as “bad” or “very bad” more frequently than normal or overweight subjects, and similar findings were obtained when the analysis was stratified by gender, although the association tended to be stronger in women (Table 2). Interestingly, opposite trends were found regarding self-rated health: in Portugal, the percentage of obese subjects rating their health as “bad” or “very bad” decreased, while the opposite trend was found in Switzerland (Table 2).

**Table 2:** Bivariate associations between body mass index categories and rating one self’s health as “bad” or “very bad”, stratified by country, gender and survey

**Survey year**	**1995-6**	**1998-9**	**2005-6**	
**Portugal, all**				
Normal	26.7	23.0	17.5	
Overweight	30.4	27.3	21.8	
Obese	41.3	36.7	32.3	
Test	238.5 ***	248.4 ***	271.8 ***	
**Portugal, men**				
Normal	23.3	21.3	15.5	
Overweight	23.6	19.3	15.9	
Obese	29.1	24.0	24.2	
Test	16.1 ***	13.3 **	47.6 ***	
**Portugal, women**				
Normal	28.7	23.9	18.7	
Overweight	35.6	33.4	27.1	
Obese	47.6	43.0	37.4	
Test	255.6 ***	333.9 ***	255.4 ***	
**Survey year**	**1992-3**	**1997**	**2002**	**2007**
**Switzerland, all**				
Normal	3.7	3.4	2.8	2.9
Overweight	4.1	5.1	4.3	4.3
Obese	6.5	6.8	7.6	9.8
Test	15.8 ***	34.6 ***	88.5 ***	180.3 ***
**Switzerland, men**				
Normal	3.4	2.9	2.6	3.2
Overweight	3.4	3.6	3.9	3.5
Obese	6.1	5.1	5.8	9.7
Test	8.28 *	6.09 *	24.3 ***	75.3 ***
**Switzerland, women**				
Normal	3.9	3.7	2.9	2.7
Overweight	5.3	7.1	4.8	5.4
Obese	6.9	8.0	9.2	9.9
Test	13.0 **	40.8 ***	86.7 ***	119.5 ***

Results are expressed as percentage of subjects rating their health as “bad” or “very bad”. Statistical analysis conducted by chi-square stratifying on country, gender and survey: *, p < 0.05; **, p < 0.01; ***, p < 0.001.

In Switzerland, an increase in the odds of rating one self’s health as “bad” or “very bad” among obese subjects was found; for instance, the 95% CI of the OR for obese subjects in 2007 did not overlap with the 95% CI for 1992-3 (Table 3). Similar albeit nonsignificant results were obtained after stratifying for gender: in men, the OR [95% confidence interval] of rating one self’s health as “bad” or “very bad” increased from 1.49 [0.97–2.30] in 1992-3 to 2.38 [1.79–3.16] in 2007; in women, the corresponding values were 1.32 [0.87–1.99] and 2.52 [1.94–3.29].

**Table 3:** Multivariate adjusted association between body mass index categories and rating one self’s health as “bad” or “very bad”, stratified by country and survey

	**Survey 1**	**Survey 2**	**Survey 3**	**Survey 4**
**Portugal**	**1995-6**	**1998-9**	**2005-6**	
Normal	1 (ref.)	1 (ref.)	1 (ref.)	
Overweight	0.94 (0.88 - 1.01)	0.97 (0.91 - 1.05)	0.98 (0.89 - 1.08)	
Obese	1.35 (1.23 - 1.48)	1.33 (1.22 - 1.46)	1.52 (1.37 - 1.70)	
**Switzerland**	**1992-3**	**1997**	**2002**	**2007**
Normal	1 (ref.)	1 (ref.)	1 (ref.)	1 (ref.)
Overweight	0.93 (0.76 - 1.14)	1.35 (1.10 - 1.65)	1.29 (1.08 - 1.54)	1.21 (1.01 - 1.44)
Obese	1.38 (1.01 - 1.87)	1.58 (1.17 - 2.14)	2.22 (1.78 - 2.78)	2.64 (2.14 - 3.26)

Results are expressed as multivariate adjusted odds ratio and (95% confidence interval). Statistical analysis adjusting for age, gender, educational level, smoking status, geographical region (Portugal) and geographical region and Swiss nationality (Switzerland).

Conversely, no clear trend was observed for Portugal, as the 95% CI for the 1995–6 and the 2005–6 surveys overlapped (Table 3). When the analysis was restricted to obese subjects, no significant trend (p = 0.23) to rate one self’s health as “bad” or “very bad” was found. Similarly, in men, the OR [95% confidence interval] of rating one self’s health as “bad” or “very bad” increased from 1.02 [0.87–1.19] in 1995–6 to 1.30 [1.08-1.56] in 2005–6; in women, the corresponding values were 1.54 [1.38–1.72] and 1.66 [1.46–1.90]. Finally, splitting the participants as <50 or ≥50 years of age showed that Portuguese obese women aged ≥50 had a lower OR of rating their health as “bad” or “very bad” than younger obese women, while no differences were found for Portuguese obese men or for Swiss participants (Table 4).

**Table 4:** Multivariate adjusted association between body mass index categories and rating one self’s health as “bad” or “very bad”, stratified by country, gender and two age groups

**Age group (years)**	**18-49**	**50+**
**Portugal, men**		
Normal	1 (ref.)	1 (ref.)
Overweight	0.69 (0.57 - 0.84)	0.81 (0.75 - 0.88)
Obese	0.89 (0.67 - 1.17)	1.05 (0.95 - 1.17)
**Portugal, women**		
Normal	1 (ref.)	1 (ref.)
Overweight	1.26 (1.12 - 1.42)	1.02 (0.96 - 1.08)
Obese	2.01 (1.75 - 2.32)	1.49 (1.38 - 1.61)
**Switzerland, men**		
Normal	1 (ref.)	1 (ref.)
Overweight	1.09 (0.87 - 1.38)	0.93 (0.78 - 1.11)
Obese	2.20 (1.56 - 3.10)	1.58 (1.25 - 1.98)
**Switzerland, women**		
Normal	1 (ref.)	1 (ref.)
Overweight	1.67 (1.32 - 2.11)	1.26 (1.09 - 1.47)
Obese	2.23 (1.59 - 3.12)	2.22 (1.85 - 2.66)

Results are expressed as multivariate adjusted odds ratio and (95% confidence interval). Statistical analysis adjusting for age, gender, geographical region, educational level, smoking status, survey year, geographical region (Portugal) and geographical region and Swiss nationality (Switzerland).

## Discussion

There are few studies that assessed the trends in the association between obesity and self-rated health [10]. In this study, we analysed the trends for Portugal and Switzerland, two small countries with differing obesity levels. Our results indicate that in Switzerland, the association between obesity and low self-rated health increased, while no such trend was found in Portugal.

Portuguese rated their health as “bad” or very bad” much more frequently than Swiss. These findings are in agreement with a previous study conducted in Europe [20] which showed consistent differences in self-health ratings, although no information regarding Portugal or Switzerland was available. Interestingly, in the European study, the regional differences persisted after adjusting for individual socio-economic data, suggesting that other factors might be at play. A study conducted in Sweden also showed no interaction between socio-economic status and obesity on self-rated health [21]. Overall, it is likely that the differences in socio-economic variables between Portugal and Switzerland might not fully account for the differences in low self-rated health, but further studies are needed to assess this point.

In both countries, obese subjects rated more frequently their health as “bad” or “very bad” than normal weight subjects, and this association remained significant after multivariate adjustment. These findings are in agreement with the literature [6,7,22], and the reasons are probably due to the fact that obesity is associated with increased objective health problems [3,4]. Also in agreement with other studies [10,23], Portuguese obese women were more likely to rate their health as “bad” or “very bad” than obese men, but no between-gender differences were found among Swiss obese. One possible reason for a lower health rating among women is a stronger social pressure to be thin [24,25], but it is rather unlikely that this could explain the differences between countries. Overall, our results indicate that obese women have a lower self-rated health than men in Portugal but not in Switzerland, and further studies are needed to better understand the differences between countries.

The prevalence of obesity in Portugal was almost twice higher than in Switzerland. Still, compared to their normal weight counterparts, Swiss obese were more likely to rate their health as “bad” or “very bad” than Portuguese. Our results speak against one of the explanations for the lack of increase in the association between obesity and self-reported overall health in the USA, *i.e.* that obese subjects feel they have a “normal”, healthy status when comparing themselves against an increasingly obese population [10,11]. An alternative explanation would be societal or cultural differences regarding body image and health, the Swiss being more concerned than other populations [26,27]. Further, several authors suggested that the association between overweight or obesity status and self-rated health varies according to gender and age [23], but the results were inconsistent. For instance, a positive association between obesity and low self-rated health was found among subjects aged 50+ in Greece [17], while in Spain this association was found among subjects aged less than 50 [18]. In this study, with the exception of Portuguese obese women, no clear differences between subjects aged <50 or ≥50 years were found for Switzerland, and these differences were cancelled after stratifying the participants in three age groups (≤44, 45 to 64 and ≥65 years, Table 5) [23]. Thus, our results indicate that, contrary to other countries, there appears to be no clear generational or age differences in rating one’s health among obese subjects in Portugal or Switzerland, but further studies are needed to better assess this point.

**Table 5:** Multivariate adjusted association between body mass index categories and rating one self’s health as “bad” or “very bad”, stratified by country, gender and three age groups

**Age group (years)**	**18-44**	**45-64**	**65+**
**Portugal, men**			
Normal	1 (ref.)	1 (ref.)	1 (ref.)
Overweight	0.64 (0.50 - 0.82)	0.82 (0.72 - 0.92)	0.86 (0.78 - 0.96)
Obese	0.83 (0.57 - 1.21)	1.08 (0.93 - 1.26)	1.19 (1.03 - 1.38)
**Portugal, women**			
Normal	1 (ref.)	1 (ref.)	1 (ref.)
Overweight	1.23 (1.06 - 1.43)	1.06 (0.97 - 1.15)	1.09 (1.00 - 1.19)
Obese	1.78 (1.47 - 2.14)	1.58 (1.43 - 1.74)	1.75 (1.57 - 1.96)
**Switzerland, men**			
Normal	1 (ref.)	1 (ref.)	1 (ref.)
Overweight	1.16 (0.89 - 1.52)	0.96 (0.77 - 1.19)	0.86 (0.67 - 1.11)
Obese	2.41 (1.62 - 3.58)	1.63 (1.23 - 2.16)	1.52 (1.08 - 2.13)
**Switzerland, women**			
Normal	1 (ref.)	1 (ref.)	1 (ref.)
Overweight	1.72 (1.32 - 2.25)	1.39 (1.13 - 1.70)	1.16 (0.95 - 1.42)
Obese	2.09 (1.38 - 3.15)	2.27 (1.76 - 2.92)	2.12 (1.66 - 2.70)

Results are expressed as multivariate adjusted odds ratio and (95% confidence interval). Statistical analysis adjusting for age, gender, geographical region, educational level, smoking status and survey. A further adjustment on geographical region (Portugal) or on geographical region and Swiss nationality (Switzerland) was performed.

This study has several limitations worth acknowledging. First, height and weight were self reported, leading to a possible underestimation of obesity levels and to an overestimation of the association between obesity and low self-rated health, as only the most obese participants will actually be considered as obese. Still, a recent study comparing self-reported and measured data suggested that the magnitude of the BMI underestimation bias decreases with time [28]. If confirmed, this would increase the difference in ORs between the earliest and the latest surveys, as the earlier values would be overestimated and the latest closer to the “true” values. It should be also noted that other studies relied on self-reported BMI data [10,29]. Second, it has been suggested that reliability of self-rated health is low, namely among disadvantaged US adults sociodemographic groups [30]. Still, self-rated health has been shown to be consistently associated with health behaviours [31,32] and outcomes in several studies, and in our multivariate analyses we systematically adjusted for educational level (a proxy of sociodemographic status). Further, as the change in self-rated health can occur either towards an improvement or a worsening, this lower reliability might increase the variance (95% confidence interval) of the association but not create a trend by itself. Third, one might question the representativeness of the Portuguese data, as in the 2005–6 survey only 15% of the sample smoked. This relatively low value might be explained by the fact that the overall smoking prevalence in Portugal is estimated at 19.7% and that Azores (smoking prevalence 24.5%) and Madeira (20.3%) were excluded from the analysis. Also, this value is close to the one reported in another Portuguese study (16.3%) [33]. Hence, we do believe that the smoking prevalence reported in our study actually reflects the true prevalence of smoking in Portugal and that the sample can be considered as representative of the population of mainland Portugal. The main strengths of this study is that it uses nationally representative samples and that the data has been collected using the same standardized methodology throughout time. Finally, the purpose of this study was to assess the trends in the associations between obesity and self-rated health separately for Portugal and Switzerland, not to compare the self-rated health status between countries. Hence, it is likely that differing health status or cultural perceptions might explain the fact that the same person with the same health would be rate her/his health as good/fair in one country and as bad in the other, but further studies are needed to assess this point.

## Conclusion

In summary, our results confirm that obese subjects tend to self-rate their health status as “bad” or “very bad” more frequently, but this lower rating does not seem to be influenced by the prevalence of obesity.

## Competing interests

The authors report no conflict of interest.

## Authors’ contributions

PMV made the statistical analysis and wrote part of the article; FP and PR revised the article for important intellectual content and wrote part of the article. PMV had full access to the data and is the guarantor of the study. All authors read and approved the final manuscript.

## Pre-publication history

The pre-publication history for this paper can be accessed here:

http://www.biomedcentral.com/1471-2458/12/588/prepub
